# Supplementary material for: A mixed methods exploration of mental health pathways and treatment as usual for young people experiencing depression in the United Kingdom
Source: Discov Health Syst. 2026 Aug 26;5(1):75. doi: 10.1007/s44250-026-00412-0 (PMC13518481; doi:10.1007/s44250-026-00412-0)
Supplement: Supplementary file 1 [file 44250_2026_412_MOESM4_ESM.pdf]

ATTEND Coding Tree

Codes

Name	Description	Files	References
Assessment related		8	15
Duty team preassessment	Relates to interaction with the duty team prior to tier 3 CAMHS assessment	0	0
In crisis		1	2
Tier 2 assessment		2	3
Tier 3 assessment	Relates to CAMHS tier 3 assessment	6	10
Time awaiting assessment	Relates to time spent between referral and tier 3 CAMHS assessment	0	0
Awaiting treatment	Relates to waiting/supportive space between assessment and the start of treatment	6	18
Clinician contact prior to treatment		3	6
Duty team monitoring	Relates to monitoring by duty team between assessment and treatment	0	0
External signposting	Relates to signposting for external support, e.g. Kooth	0	0

Name	Description	Files	References
Mental health resources	Relates to provision of mental health resources and literature between assessment and the start of treatment	1	1
Treatment wait time	Relating to amount of time spent waiting between assessment and first-line treatment	5	6
Clinician and CAMHS factors		11	42
Assessments		1	1
Buddhist psychotherapist		1	2
Case management		2	4
CBT therapist		3	3
Consultation		1	1
CWP		2	2
Family therapy		1	2
Pandemic related pressures		1	3
Part of tier 3 CAMHS		3	3
Pressures on CAMHS		3	5

Name	Description	Files	References
Psychologist		2	2
Psychotherapy		1	1
Supervision		3	3
Systemic work		2	2
Team management		2	2
Training		1	1
Treating trauma		1	2
Types of presentation		3	3
Discharge	Relates to discharge from CAMHS	7	16
Follow-up contact		2	3
Referral to other services		3	5
Self-help resources		2	2
Entry into service and referral	Relating to referral into CAMHS service	7	9
Ineligible		0	0

Name	Description	Files	References
17 IAPT	Referral to 17+ IAPT service	0	0
CWP	Relates to referral to CWP/first line CAMHS	0	0
School MH team	Relates to referral to school MH team	0	0
Tier 4	Relates to referral to tier 4 CAMHS/N&S team	0	0
Screening and intake	Relating to screening and intake for tier 2/3/4 CAMHS	4	4
MAC intervention		5	29
Advantages		0	0
Applied to other areas of life		1	1
Confidence in groups		1	2
Effective		4	8
Engagement		1	1
Enjoyable		1	2
Expressing needs		1	1
Novelty		1	1

Name	Description	Files	References
Second-line treatment option		2	2
Seeing other people in similar situations		2	3
Disadvantages		0	0
Additional appointments		3	5
Difficult		2	2
Falling asleep		1	1
Group work		1	2
Homework		1	1
Lack of engagement		4	6
Misconceptions about mindfulness		2	2
Not helping		1	2
Triggered anxiety		2	3
Modifications which support		1	4

Name	Description	Files	References
Parent attendance		3	6
Recruitment for study		2	2
Young person's attitude to MAC		5	14
Patient factors		9	43
Diagnoses and presentation		7	21
Length of illness		5	5
Past trauma		1	5
Personality and attitude		2	2
Pressures on treatment		1	2
Risk		2	4
Stressors and social factors		2	4
Treatment for MAC participants during ATTEND		10	64
Behavioural activation		1	5
CBT		1	4

Name	Description	Files	References
Check in sessions		4	7
Compassion techniques		1	1
DBT		2	3
ELSA intervention		1	1
Follow up		1	1
General therapeutic work		5	12
Inpatient treatment		1	2
Medication		3	5
Mindfulness		1	1
No other treatment		2	3
Patient attitude to treatment		1	1
Relapse prevention		1	1
Risk management		2	2
Support with MAC treatment		1	5

Name	Description	Files	References
Systemic work		3	4
Waiting list		3	4
Working with other adults		2	2
Treatment for TAU participants during ATTEND		2	12
General therapeutic work		2	5
Medication		2	2
Mindfulness		1	1
Other study		1	1
Patient attitude to treatment		1	1
Systemic work		1	2
Treatment recording sheet		4	5
Advantages		2	2
Captures useful info		2	2
Fits with work done		3	3

Name	Description	Files	References
Index useful		1	2
Manageable		6	9
Provided insights		2	3
Disadvantages		1	1
Confusion of how to record treatment frequency		1	1
Confusion over whether to include treatment prior to ATTEND		4	8
Doesn't fit with type of work done		3	5
Laborious		4	5
Misses something		4	5
Not useful		2	3
Wasn't able to look at it		1	1
Modifications		2	2
Treatments prior to ATTEND study	Refers to treatments provided before the study began	11	123

Name	Description	Files	References
ACT		0	0
Art therapy		1	1
Behavioural activation		2	5
BPI		0	0
CBT	Refers to CBT as a first line treatment	6	15
CFT		1	1
CWP treatment		2	3
Day patient treatment		1	1
DBT		3	3
Family therapy	Relates to family therapy as a first-line treatment	3	7
General therapeutic work		9	15
Gradual exposure		1	1
Inpatient treatment		2	3
IPT		1	2

Name	Description	Files	References
LiCBT		1	8
Link to other services	Refers to linking to other agencies e.g. social care	0	0
Managing safety		5	7
Medication		5	7
Mindfulness		2	3
Patient attitude to treatment		3	7
Psychodynamic psychotherapy		0	0
Psychoeducation		1	1
Response to treatment		4	6
Second line treatment	Relates to provision of a second line treatment	4	8
STPP		0	0
Systemic work		3	6
Tier 4 CAMHS	Relates to referral to national and specialist team (tier 4 CAMHS)	0	0
Trauma work		1	3

Name	Description	Files	References
Virtual treatment		1	1
Working with other adults		6	7
